# Immune dysregulation is associated with symptom dimensions and cognitive deficits in schizophrenia: accessible evidence from complete blood count

**DOI:** 10.1186/s12888-023-05430-3

**Published:** 2024-01-12

**Authors:** Lina Zhou, Xiancang Ma, Wei Wang

**Affiliations:** Department of Psychiatry, The First Affiliated Hospital of Xi’an Jiaotong University, 277 West Yanta Road, Xi’an, Shaanxi P.R. China

**Keywords:** Schizophrenia, Dysregulated immunity, Cognitive deficits, Complete blood count, Symptoms

## Abstract

**Background:**

Schizophrenia (SCZ) is a psychotic disorder with an unknown pathogenesis accompanied by varying degrees of cognitive deficits. Recent studies have shown that immune dysregulation plays an important role in developing symptoms and cognitive deficits in SCZ. This study aimed to determine the complete blood count (CBC), including white blood cells, neutrophils, monocytes, lymphocytes, platelets, neutrophil-lymphocyte ratio (NLR), platelet-lymphocyte ratio (PLR), and monocyte-lymphocyte ratio (MLR), in patients with SCZ and explore their correlations with SCZ symptom dimensions and cognitive function.

**Methods:**

Seventy-four patients with SCZ and 57 age- and sex-matched healthy controls with available demographic and clinical information were recruited for this study. Blood samples were collected, and symptom dimensions and cognitive function were evaluated using the Positive and Negative Syndrome Scale (PANSS) and MATRICS Consensus Cognitive Battery (MCCB) separately.

**Results:**

Our results demonstrate that SCZ patients showed higher monocyte counts, PLR, MLR, and worse performance in the total MCCB than healthy controls. Neutrophil and lymphocyte counts and NLR were positively related to symptom severity and negatively related to depressive symptoms. White blood cell (WBC) count, monocyte count, and MLR were positively correlated with cognitive performance in patients with SCZ.

**Conclusion:**

In summary, this study suggests that cognitive deficits and symptom severity in patients were associated with dysregulation of immunity. Moreover, we found that WBC could be used as a marker for symptom severity and cognitive deficits in SCZ and that neutrophils are more closely related to the former and monocytes to the latter. We hope that clinicians will pay more attention to dysregulated immunity in patients with SCZ in the future.

## Introduction

SCZ (SCZ) is a psychotic disorder with a high incidence of morbidity and social burden [1]. However, the pathogenesis of SCZ remains unclear. A growing body of evidence suggests that dysfunctional immune function is involved in the pathogenesis of SCZ [2, 3]. Cognitive deficit is one of the core symptoms of SCZ, Ribeiro-Santos et al. reported that inflammation was associated with worst cognitive performance in patients with SCZ [4]. Thus, it reveals that immune dysregulation plays an important role in the pathophysiology of SCZ and is involved in the mechanism of cognitive deficits [5, 6].

White blood cells (WBC), a major indicator of Complete blood count (CBC), including neutrophils, lymphocytes and monocytes, are the markers of inflammation in the body. Neutrophil/lymphocyte ratio (NLR), platelet/lymphocyte ratio (PLR), and monocyte/lymphocyte ratio (MLR) are also indicators of inflammation. Marazziti et al. [7] pointed out that NLR, PLR, and MLR seem promising tools for the economical and easy detection of inflammatory system activation. These views can easily and quickly assess mental disorders in patients with inflammatory changes and have now been used in patients with SCZ. For example, a meta-analysis found increased total and differential WBC counts in patients with SCZ, such as increased monocyte and neutrophils [8]. Zhu et al. [9] found that platelet and lymphocyte counts were significantly lower, while NLR and MLR were significantly higher in patients with SCZ than in healthy controls. Özdin and Böke [3] compared the complete blood counts (CBC) of 105 patients diagnosed with SCZ during the relapse and remission periods and found that the NLR, PLR, and MLR of the patients during the relapse period were significantly higher than those in the control group. These results indicate that CBC indicators, including total and differential WBC counts, NLR, PLR, and MLR, can be used as convenient and economical inflammatory indicators to effectively reflect inflammatory changes in patients with SCZ.

Since peripheral inflammation might be related to cognitive deficits in SCZ [10], it is of great significance to explore the relationship between total and differential WBC counts, NLR, PLR, symptom dimensions, and cognitive function in patients with SCZ and further explore the mechanism of SCZ. Moreover, few studies have explored the relationship between the MATRICS Consensus Cognitive Battery (MCCB) and inflammatory markers as tools to assess cognitive function in patients with SCZ. Based on the above background, this study aimed to explore the changes in CBC in patients with SCZ and their relationship with symptom dimensions and cognitive function. This study had two hypotheses: (1) CBC would be changed in patients with SCZ compared to healthy controls, and (2) CBC would be related to symptom dimensions and cognitive performance in patients with SCZ.

## Methods

### Subjects

All patient subjects were inpatients recruited from the Department of Psychiatry at the First Affiliated Hospital of Xi’an Jiaotong University. The inclusion criteria of patients were as follows: (1) Patients were diagnosed with SCZ according to the Fifth Vision of American Diagnostic and Statistical Manual of Mental Disorders (DSM-V); (2) Age were between 18 and 50; (3) Total Positive And Negative Syndrome Scale (PANSS) ≥ 60; (4) Subjects were without autoimmune disease, chronic inflammation, acute infection or antibiotics in the past two weeks; (5) Subjects were without severe somatic disease; (6) There were no other diseases that meet the diagnostic criteria of DSM-V at present. Healthy controls were recruited from nearby schools and communities, and their inclusion criteria were as follows: (1) subjects who did not meet any diagnostic criteria for DSM-V; (2) aged between 18 and 50 years; (3) subjects without autoimmune disease, chronic inflammation, acute infection, or application of antibiotics in the past two weeks; and (4) subjects without severe somatic disease.

In addition to the above inclusion criteria, all subjects were required to be able to understand and complete all cognitive tasks. All subjects were of Han Chinese ethnicity, residing in the Shaanxi area, received a detailed introduction to the study, and provided written informed consent. This study was approved by the Ethics Committee of the First Affiliated Hospital of Xi’an Jiaotong University (approval no. XJTU1AF2015LSL-079).

### Clinical measure

All SCZ patients were assessed by an experienced psychiatrist using the Chinese version of the Positive and Negative Syndrome Scale (PANSS). PANSS was analysed using a five-factor model including positive symptoms (P1-P7), negative symptoms (N1-N7), cognitive symptoms (P2, N5, N7, G10, and G11), excitement symptoms (P4, P7, G4, G8, and G14), and depressive symptoms (G2, G3, and G6) [11].

### Cognitive tasks

The Chinese version of the MCCB has satisfactory psychometric properties, including high test-retest reliability, high internal consistency, acceptable concurrent validity, and good discriminant validity [12, 13]. In this study, the following six cognitive tasks of MCCB were selected:1) The symbol coding test (SCT) and category fluency (SF) were used to evaluate the speed of processing information; 2) The Hopkins verbal learning test (HVLT) was a test used to assess verbal learning and memory; 3) The brief visuospatial memory test (BVMT) was a test of visual learning and visual memory; 4) The space span test (SST) was a test for working memory; 5) The neuropsychological assessment battery (NAB) was used to test reasoning and problem-solving abilities.

### Complete blood count

Blood samples were obtained between 06:00 am and 9:00 am on the day of enrolment, following an overnight fast. Overnight fasting was also monitored. Immediately after collecting blood samples, the complete blood count was determined using a Sysmex HST-201 Automated Haematology Analyser (Sysmex, USA). The absolute numbers of white blood cells, platelets, neutrophils, lymphocytes, and monocytes were measured. NLR, PLR, and MLR were calculated as the neutrophil/lymphocyte, platelet/lymphocyte, and monocyte/lymphocyte ratios, respectively.

### Data analysis

SPSS (version 24.0; SPSS Inc., Chicago, Illinois, USA) was used for the statistical analysis. Categorical variables were analysed using the chi-squared test. Continuous variables (mean ± standard deviation) were determined to determine whether they conformed to a normal distribution using the Kolmogorov-Smirnov test. Normally distributed parameters were tested using a two-tailed independent sample T-test, and non-normally distributed parameters were analysed using a non-parametric test, namely the Mann–Whitney U test. Group comparisons of cognitive function and CBC were performed using a one-way analysis of variance. Correlations among PANSS, cognitive performance, and CBC were analysed using multiple linear regression. All statistical tests were two-tailed, and differences were considered significant at *p* < 0.05.

## Results

### Demographic, clinical information, and cognitive performance

The demographic, clinical, and cognitive characteristics of all subjects are listed in Table 1. There were no significant differences in sex, age, BMI, and number of married individuals and smokers between the groups. Patients with SCZ had significantly fewer years of education than controls. All MCCB tasks in this study were found to have worse performance in patients with SCZ than in healthy controls, including the SCT, HVLT, SST, NAB, BVMT, and SF. Among patients with SCZ, 78.38% had a primary episode, and 21.62% had a recurrent episode. Their average illness duration was (14.51 ± 19.44) months, the equivalent drug dose of olanzapine was (6.36 ± 4.62) mg/d, and the average PANSS was (90.59 ± 14.19).

**Table 1:** Demographic, clinical information, and cognition performance of healthy controls and SCZ patients

	HC (n = 57)	SCZ (n = 74)	Statistic	*p*
Gender				
Male, n (%)	33(57.89)	42(56.76)	0.017	0.896
Female, n (%)	24(42.11)	32(43.24)		
Age, year, M ± SD	27.39 ± 6.06	27.26 ± 7.39	-0.419	0.676
BMI, kg/m^2^, M ± SD	21.60 ± 2.60	20.76 ± 2.67	1.791	0.076
Smoking				
Yes, n (%)	12(21.05)	16(21.62)	0.006	0.937
No, n (%)	45(78.95)	58(78.38)		
Marriage				
Yes, n (%)	45(78.95)	53(71.62)	0.917	0.338
No, n (%)	12(21.05)	21(28.38)		
Education, year, M ± SD	15.72 ± 1.57	12.26 ± 3.35	7.851	**< 0.001**
Primary or recurrent				
Primary episode, n (%)	-	58(78.38)	-	-
Recurrent episode, n (%)	-	16(21.62)		
Illness duration, month, M ± SD	-	14.51 ± 19.44	-	-
Equivalent drug dose, mg/d, M ± SD	-	6.36 ± 4.62	-	-
Total PANSS	-	90.59 ± 14.19	-	-
Positive symptom	-	12.51 ± 3.63	-	-
Negative symptom	-	19.55 ± 5.65	-	-
Cognitive defect	-	10.36 ± 3.22	-	-
Excited symptom	-	10.36 ± 3.35	-	-
Depressive symptom	-	8.61 ± 3.28	-	-
Total MCCB	257.00 ± 54.46	174.57 ± 69.51	54.400	**< 0.001**
SCT	49.46 ± 16.00	37.65 ± 13.44	21.038	**< 0.001**
HVLT	41.39 ± 11.34	30.43 ± 14.35	22.402	**< 0.001**
SST	33.93 ± 14.59	21.93 ± 12.39	25.854	**< 0.001**
NAB	43.72 ± 17.18	25.35 ± 18.76	33.183	**< 0.001**
BVMT	44.47 ± 15.34	30.82 ± 16.50	23.411	**< 0.001**
SF	44.04 ± 15.66	28.38 ± 16.22	30.907	**< 0.001**

### CBC and its correlation with clinical symptoms

The complete blood counts of all the subjects are listed in Table 2. Compared to healthy controls, patients with SCZ showed higher monocyte counts, PLR, and MLR. Further grouping by sex revealed that the monocyte count, PLR, and MLR were significantly higher in male patients with SCZ than in male controls. In contrast, the PLR was significantly higher in female patients than in female controls. Linear regression analysis explored the correlation between the CBC count and clinical symptoms in patients with SCZ. After adjusting for sex, age, BMI, smoking, years of education, course of disease, and drug equivalent dose, it was found that the total PANSS score was positively correlated with WBC count, and negative and depressive symptoms were all positively correlated with WBC count, neutrophil count, and NLR. Moreover, excitement symptoms correlated with lymphocyte counts, as shown in Table 3.

**Table 2:** Complete blood cell count of healthy controls and SCZ patients

	HC			SCZ			*p*		
	Total	Male	Female	Total	Male	Female	Total	Male	Female
WBC, 10^9^/L	6.04 ± 1.21	6.39 ± 1.05	5.56 ± 1.27	6.69 ± 2.47	6.82 ± 2.09	6.53 ± 2.92	0.070	0.290	0.134
Plt, 10^9^/L	225.32 ± 60.39	209.42 ± 52.41	247.17 ± 64.79	211.64 ± 55.26	212.21 ± 43.51	210.89 ± 68.45	0.180	0.802	0.050
Neu, 10^9^/L	3.46 ± 1.06	3.69 ± 0.94	3.16 ± 1.16	3.63 ± 1.47	3.72 ± 1.46	3.51 ± 1.50	0.482	0.928	0.340
Lym, 10^9^/L	2.12 ± 0.49	2.22 ± 0.48	1.98 ± 0.47	2.02 ± 0.52	2.10 ± 0.59	1.93 ± 0.40	0.300	0.335	0.689
Mon, 10^9^/L	0.32 ± 0.08	0.34 ± 0.08	0.29 ± 0.09	0.37 ± 0.15	0.41 ± 0.18	0.31 ± 0.09	**0.030**	**0.036**	0.357
NLR	1.72 ± 0.65	1.75 ± 0.58	1.68 ± 0.74	1.95 ± 1.28	1.99 ± 1.51	1.90 ± 0.93	0.209	0.381	0.337
PLR	71.69 ± 30.24	60.19 ± 21.71	87.50 ± 33.47	109.62 ± 35.26	108.54 ± 37.01	111.04 ± 33.34	**< 0.001**	**< 0.001**	**0.012**
MLR	0.16 ± 0.05	0.16 ± 0.05	0.15 ± 0.06	0.19 ± 0.08	0.20 ± 0.10	0.17 ± 0.06	**0.010**	**0.011**	0.380

**Table 3:** Correlations between CBC and PANSS symptoms in patients with schizophrenia

	P	N	C	E	D	Total PANSS
WBC	0.690	**0.025**	0.136	0.706	**0.002**	**0.004**
Plt	0.892	0.418	0.601	0.518	0.805	0.501
Neu	0.225	**0.033**	0.964	0.448	**0.032**	0.226
Lym	0.558	0.872	0.747	**0.037**	0.799	0.908
Mon	0.896	0.087	0.864	0.450	0.136	0.224
NLR	0.339	**0.035**	0.535	0.603	**0.020**	0.085
PLR	0.715	0.869	0.774	0.166	0.400	0.845
MLR	0.994	0.068	0.890	0.149	0.067	0.150

Variables such as sex, age, BMI, smoking, marriage, education level, course of disease, and drug equivalent dose were adjusted

### Correlations between CBC and cognitive performance

The correlations between CBC and cognitive performance in healthy controls and patients are presented in Table 4. The results showed that after adjusting for relevant variables, including sex, age, BMI, smoking, marital status, and education level, WBC count and MLR were associated with multiple cognitive function scores in the control group. However, this correlation decreased in the SCZ group. Specifically, after adjusting for sex, age, BMI, smoking, marital status, education level, disease duration, and drug equivalent dose, there were positive correlations between the WBC count and SCT, HVLT, SST, SF, monocyte count, MLR, and SCT.

**Table 4:** Correlations between CBC and cognitive performance in healthy controls and patients with schizophrenia

	Total MCCB		SCT		HVLT		SST		NAB		BVMT		SF	
	HC	SCZ	HC	SCZ	HC	SCZ	HC	SCZ	HC	SCZ	HC	SCZ	HC	SCZ
WBC	**< 0.001**	0.137	0.892	**0.004**	**0.029**	**0.029**	**< 0.001**	**0.012**	**0.002**	0.214	0.053	0.322	**0.045**	**0.030**
Plt	0.210	0.667	0.054	0.404	0.387	0.665	0.645	0.987	0.892	0.886	0.297	0.749	0.280	0.858
Neu	**0.001**	0.478	0.871	0.137	**0.016**	0.333	**0.003**	0.341	**0.002**	0.378	0.058	0.875	0.093	0.112
Lym	0.123	0.945	0.197	0.548	0.912	0.509	0.154	0.782	0.916	0.526	0.937	0.577	0.489	0.864
Mon	0.955	0.259	0.463	**0.010**	0.838	0.728	0.972	0.755	0.363	0.502	0.596	0.068	0.467	0.549
NLR	0.132	0.430	0.481	0.163	0.061	0.466	0.211	0.358	0.055	0.162	0.296	0.811	0.441	0.158
PLR	**0.039**	0.896	0.300	0.504	0.100	0.487	**0.010**	0.888	**0.003**	0.467	0.252	0.701	0.577	0.908
MLR	0.527	0.383	0.961	**0.028**	0.960	0.543	0.364	0.770	0.348	0.969	0.654	0.092	0.333	0.513

Variables such as gender, age, BMI, smoking, marriage, and education level were adjusted in healthy controls. Course of disease and drug equivalent dose. In addition, the schizophrenia patient group was additionally corrected for disease duration and drug equivalent dose

## Discussion

One of the pathogeneses of SCZ is dysregulated immunity, which is particularly involved in cognitive deficits. Therefore, it is necessary to explore changes in inflammatory indicators and their relationship with cognitive function in patients with SCZ. The complete blood count (CBC) is simple, readily available, and accurately reflects the indicators of body inflammation, and has been used to discover that there is a difference in the inflammatory response between SCZ and normal individuals [3]. However, no study has examined the relationship between CBC and cognitive deficits in patients with SCZ. Our results show an inflammatory difference between SCZ and normal individuals, and SCZ patients have higher monocyte count, PLR, and MLR. WBC, neutrophil, lymphocyte counts and NLR are related to symptom severity and negative and depressive symptoms in patients with SCZ. Furthermore, we assessed cognitive function in SCZ using the MCCB and found that patients with SCZ had comprehensive cognitive deficits involving the speed of processing information, verbal learning and memory, visual learning and memory, working memory, reasoning, and problem-solving abilities. We also found positive correlations between WBC count, monocyte count, MLR, and cognitive performance in healthy controls and patients. These results prove that immune dysregulation is related to the symptom dimensions and cognitive deficits in SCZ.

Steen et al. [14] suggested that genetic factors associated with WBC counts are associated with the risk of SCZ. A bidirectional two-sample Mendelian randomisation study [15] showed that SCZ was associated with an elevated WBC count (i.e. higher WBC, lymphocyte, and neutrophil counts). These findings suggest that inflammatory changes related to WBC may be involved in the pathogenesis of SCZ. In addition, the activation of microglia plays a vital role in the inflammatory immune mechanism of SCZ, and platelets are important in the activation of microglia [16]. Peripheral monocytes are thought to have many commonalities with microglia [17], which may result in increased PLR and MLR in SCZ. Similar to previous findings, our study found that patients with SCZ had higher monocyte counts, PLR, and MLR. In addition to that, numerous previous studies have shown that females generally have a higher innate immune response than males. Schneider et al. [18] and our study found the activation of innate immunity in male rather than female SCZ patients. This may be because females have a higher cortisol response than males, resulting in an imbalance between the HPA axis and the immune system [18]. Collectively, these results suggest that changes in inflammation are more closely associated with SCZ pathogenesis in male patients.

Šagud et al. [19] reported that most negative symptoms were weakly to moderately positively correlated with blood cell inflammatory ratios, namely, between the NLR and MLR with the PANSS negative scale. Zhou et al. [20] found that NLR was negatively associated with the negative symptoms of the Brief Psychiatric Rating Scale. A study including 137 first-episode psychosis and 81 healthy controls found that the neutrophil count was associated with reduced grey matter and enlarged ventricles in first-episode psychosis, providing indirect evidence that neutrophils are involved in psychopathology [21]. The relationship between NLR and depression severity in patients with depression [22] and NRL and psychotic depression [23] has been previously reported, and depression has many similarities with negative symptoms, prompt NLR, negative symptoms of SCZ, and depressive symptoms. In the present study, we also found that WBC count, neutrophil count, and NLR were significantly related to PANSS negative and depressive scales, suggesting that neutrophil-mediated immune responses in the brain play an important role in the clinical manifestations of schizophrenia, especially negative and depressive symptoms.

In this study, we also found that WBC was associated with several cognitive functions, including the speed of processing information, verbal learning and memory, and working memory, suggesting that cognitive deficits in SCZ are associated with an increase in overall immune levels. Ribeiro-Santos et al. [4] suggested that microglial activation, monoaminergic imbalance, brain abnormalities, and the kynurenine pathway are possible mechanisms underlying cognitive impairment in SCZ. As mentioned earlier, monocytes play an important role in microglia activation. Mazza et al. [24] suggested that monocyte count could be considered an indirect marker of microglial activation in the central nervous system, and we also found that monocyte counts and MLR were associated with cognitive deficits in patients with SCZ. Recent studies have also found that monocytes may have negative effects on brain structure and cognition in patients with SCZ [25]. The MLR of cerebrospinal fluid was associated with verbal fluency [26], which demonstrates that the increase in mononuclear cells is activated by microglial cells, which is one of the most important aspects of cognitive deficits in SCZ and is mainly related to the speed of processing information represented by SCT.

Our study has some limitations. First, we did not simultaneously examine other inflammatory markers to assess inflammatory changes in patients with SCZ, although the NLR and MLR have been identified as markers of peripheral inflammation in many studies. Second, considering that the acute stage of SCZ is more representative, all selected patients were in the acute stage of SCZ, which tends to show poor cooperation. Therefore, only six cognitive tasks were selected for the MCCB, although these tasks broadly reflect cognitive function.

In conclusion, we preliminarily explored the relationship among symptom dimensions, cognitive function, and CBC in patients with SCZ and found that cognitive deficits and symptom severity were associated with dysregulated immunity. Moreover, we found that WBC could be used as a marker for symptom severity and cognitive deficits and that neutrophils are more closely related to the former and monocytes to the latter. We hope that clinicians will pay more attention to dysregulated immunity in patients with SCZ in the future.

## Data Availability

The datasets used during the current study available from the corresponding author on reasonable request.

## Abbreviations

HC: Healthy controls
SCZ: Schizophrenia
M ± SD: Mean ± standard deviation
BMI: Body mass index
PANSS: Positive and Negative Syndrome Scale
MCCB: MATRICS Consensus Cognitive Battery
SCT: Symbol coding test
SF: Category fluency
HVLT: Hopkins verbal learning test
BVMT: Brief visuospatial memory test
SST: Space span test
NAB: Neuropsychological assessment battery
WBC: White blood cell
Plt: Platelets
Neu: Neutrophil
Lym: Lymphocyte
Mon: Monocyte
NLR: Neutrophil/lymphocyte ratio
PLR: Platelet/lymphocyte ratio
MLR: Monocyte/lymphocyte ratio
P: Positive symptom
N: Negative symptom
C: Cognitive defect
E: Excited symptom
D: Depressive symptom
